# Detection of Gait Modes Using an Artificial Neural Network during Walking with a Powered Ankle-Foot Orthosis

**DOI:** 10.1155/2016/7984157

**Published:** 2016-12-13

**Authors:** Mazharul Islam, Elizabeth T. Hsiao-Wecksler

**Affiliations:** Mechanical Science and Engineering, University of Illinois at Urbana-Champaign, Urbana, IL 61801, USA

## Abstract

This paper presents an algorithm, for use with a Portable Powered Ankle-Foot Orthosis (i.e., PPAFO) that can automatically detect changes in gait modes (level ground, ascent and descent of stairs or ramps), thus allowing for appropriate ankle actuation control during swing phase. An artificial neural network (ANN) algorithm used input signals from an inertial measurement unit and foot switches, that is, vertical velocity and segment angle of the foot. Output from the ANN was filtered and adjusted to generate a final data set used to classify different gait modes. Five healthy male subjects walked with the PPAFO on the right leg for two test scenarios (walking over level ground and up and down stairs or a ramp; three trials per scenario). Success rate was quantified by the number of correctly classified steps with respect to the total number of steps. The results indicated that the proposed algorithm's success rate was high (99.3%, 100%, and 98.3% for level, ascent, and descent modes in the stairs scenario, respectively; 98.9%, 97.8%, and 100% in the ramp scenario). The proposed algorithm continuously detected each step's gait mode with faster timing and higher accuracy compared to a previous algorithm that used a decision tree based on maximizing the reliability of the mode recognition.

## 1. Introduction

Everyday walking is generally not only limited to level, overground walking but also involves ascending and descending stairs and ramps. Lower limb joint kinematics and kinetics change with different types of walking environments, or gait modes [1]. Therefore, the ability to recognize and control for different gait modes when using powered lower limb prostheses or orthoses must be addressed if the devices are to be used beyond treadmill walking or walking around a clinic or laboratory.

Several studies have explored gait mode recognition [1–20]. Most were for prosthetic devices [2–11, 17–20]. Some used manual switching schemes to deal with changing gait modes during walking. Otto Bock [4] demonstrated an approach where a user manually switches modes using Ottobock's C-Leg by tapping the heel. Au et al. proposed two finite state controllers to classify between level ground and stair descent mode using electromyography signals measured from intentionally activated residual muscles in the amputated limb [5]. This approach also needed a large number of sensor signals and could not detect stair ascent mode. These algorithms were not autonomous and needed user's input [4, 5].

Other studies have used autonomous systems based on a variety of electromechanical sensors for detecting gait modes. A number of methods (using socket interface forces, ankle angle, or knee angle as input signals) were studied by the group at Vanderbilt University for use with their lower limb prosthesis [6–11]. A *k*-nearest neighbor algorithm to classify different gait modes was used; however, in that study, three different walking speeds were considered as the different gait modes and not the changes in walking environment [6]. In a separate study, principle component analysis with Gaussian mixture models was used for gait mode recognition; two modes (standing and walking mode) were considered as gait modes [7–9]. A supervisory intent classifier combined with a mid-level controller based algorithm to switch modes was also examined [11]. These schemes were capable of detecting different phases only during level ground walking (Phase 1: stance flexion/extension; Phase 2: preswing; Phase 3: swing flexion; Phase 4: swing extension). Another common approach for gait mode recognition involved using inertial measurement units (IMUs) or other sensors [12–16]. Zhang et al. [12] developed an algorithm to predict upcoming terrain height using a large number of sensors (laser sensor and four IMUs). Wang et al. [13] also detected the locomotion mode recognition by using similar sensor signals (laser sensor and IMUs). These algorithms needed heavy computation to deal with lots of data collected from the multiple different sensors. Coley et al. [17] used a miniature gyroscope attached to the shank to detect level ground and stair ascent modes. Being a noncausal algorithm, this procedure could not be implemented in real-time and had to be implemented during postprocessing of the data since future input is needed for the algorithm. Jang et al. [18] measured hip joint angles of both legs and signals from IMUs at the moment of foot contact to recognize level ground, stair ascent, or stair descent by using a hip exoskeleton. This algorithm had a one-step delay, such that the first step transitioning into a new mode was always unrecognized.

Various researchers have used EMG signals from residual muscles in the amputated limb for detecting intent recognition using prosthetic devices [19–23]. Young et al. designed a dynamic Bayesian network for processing both mechanical (velocity and position) and EMG sensors for intent recognition [19, 20]. Miller et al. performed walking mode classification by using linear discriminant analysis (LDA) and support vector machine (SVM) on EMG signals for transtibial amputees [21]. A similar approach was also used by Huang et al. [22, 23]. However, EMG signals collected from patients with lower limb impairment due to neuromuscular deficits may likely be unreliable or nonexistent. Thus, the use of EMG signals for gait mode recognition of powered orthoses used on such patient populations may not be appropriate.

**Figure 1:**
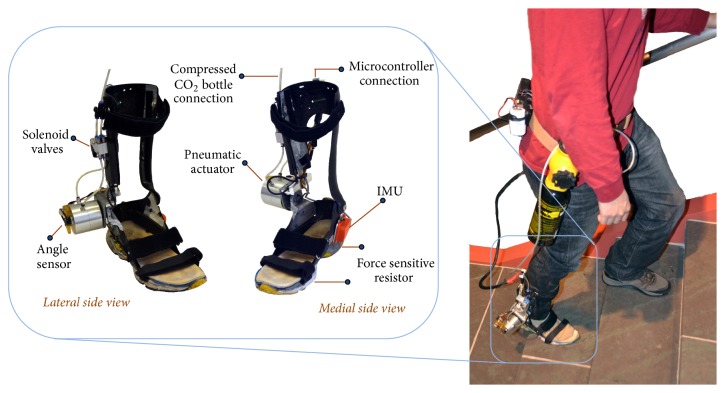
The pneumatic Portable Powered Ankle-Foot Orthosis (PPAFO).

Li and Hsiao-Wecksler [16] proposed an algorithm to recognize gait modes by using the Portable Powered Ankle-Foot Orthosis (PPAFO). The PPAFO can provide powered dorsiflexor or plantarflexor torque assistance to the ankle joint using a pneumatic system and waist-worn tank of compressed carbon dioxide (Figure 1). The algorithm used the real-time vertical position and orientation of the foot using an IMU on the PPAFO and also foot-ground contact information from force sensitive resistor (FSR) sensors under the heel and toe. This algorithm used training data to calculate optimal threshold values for different stair heights or ramp grades; then during test cases, the algorithm checked the height between two consecutive strides and foot orientation to detect changes in gait mode. As a consequence of this comparison approach, limitations of this algorithm were dependent on multiple trained stair heights or ramp grades and a one-step delay in mode recognition.

In summary, existing gait mode recognition schemes have shortcomings of not being autonomous [4, 5], delay in detection of a new mode until the next step [16, 18], difficulty of implementation in real-time [6–10, 17], dependency on trained stair heights [16], or need for a large number of sensors [12–15, 19, 20]. There are currently no reliable gait mode recognition algorithms available which can detect all of the modes without long delays and without the use of a large number of sensors. In this study, we proposed an artificial neural network (ANN) based algorithm to detect gait modes automatically. This algorithm used data from a single IMU, with fail-safe operation using two foot switches. Two of the greatest advantages of ANNs are to approximate an arbitrary function and also estimate a nonlinear model after learning from observed data [24]. For this reason, an ANN was used to estimate gait modes in this paper. We hypothesized that this approach would detect different gait modes (level ground, ascent of stairs or ramps, and descent of stairs or ramps) with higher accuracy and less delay than the previous autonomous algorithm that was proposed in Li and Hsiao-Wecksler [16].

## 2. Method

### 2.1. Proposed Approach

A supervised learning recognition approach was developed using an artificial neural network (ANN) for detecting the gait modes. This approach used a multilayer feed-forward ANN of one hidden layer and one output layer. Vertical foot velocity and foot segment angle were used as the inputs. Training was done to determine model parameters; then the approach was applied to gait data collected on five healthy young adults. Success rate of the proposed algorithm was evaluated and compared with the previously developed algorithm by Li and Hsiao-Wecksler [16].

#### 2.1.1. Design of Artificial Neural Network

**Figure 2:**
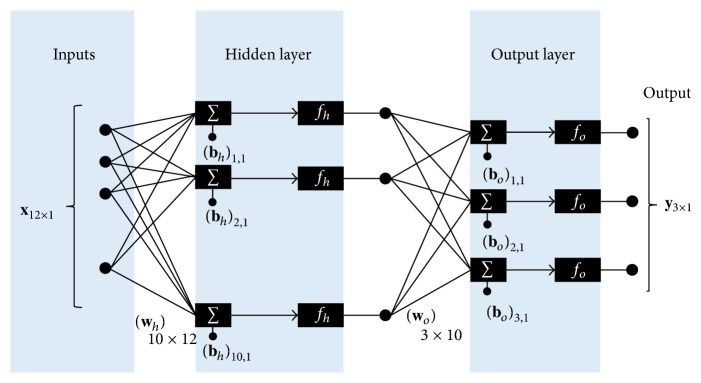
Artificial neural network structure for gait mode recognition.

A feed-forward, multilayer, artificial neural network [24] was used to perform gait mode recognition (Figure 2). In the input layer, there was an input vector which consisted of two sensor signals (foot velocity and segment angle) with six tapped delays each, for a total of 12 elements. To compensate for inherent sensor noise, tapped delays were used in this study [24]. We conducted a preliminary study to determine the appropriate number of tapped delays to use. Six tapped delays were chosen because the performance of the ANN improved and then plateaued for more than five tapped delays. The hidden layer had 10 neurons and used log-sigmoid activation functions. The output layer had three neurons and used a linear activation function, which provided a vector with three elements. As a network with one hidden layer with log-sigmoid activation function and one output layer with linear activation function is considered as a universal function approximation [24], this study used this configuration.

Log-sigmoid functions were used as the activation function in the hidden layer. So the output of the hidden layer, **a**_*h*_, can be found from

(1) $$\begin{array}{c} a_{h}=f_{h}\left(\;w_{h}x+b_{h}\;\right). \end{array}$$

Here, **x** is the vector of inputs and (**w**_*h*_, **b**_*h*_) are the weights and biases of the hidden layers. *f*_*h*_(*n*) can be expressed as

(2) $$\begin{array}{c} f_{h}\left(\;n\;\right)=\frac{1}{e^{-n}+1}. \end{array}$$

The output layer provided the vector **y**(*t*) which can be found using

(3) $$\begin{array}{c} y\left(\;t\;\right)=f_{o}\left(\;w_{o}a_{h}+b_{o}\;\right), \end{array}$$

where (**w**_*o*_, **b**_*o*_) are the weights and biases of the output layer. *f*_*o*_(*n*) is the output layer activation function, which was defined as a linear activation function:

(4) $$\begin{array}{c} f_{o}\left(\;n\;\right)=n. \end{array}$$

The parameters (**w**_*h*_, **b**_*h*_) and (**w**_*o*_, **b**_*o*_) will be estimated for finding the output of the multilayer neural network for any input **x**(*t*) and training is needed to evaluate these parameters. The gait modes, observed during the collection of training data, were used as the target values (**t**_*i*_) for training the ANN. During the training, we minimized the cost function *E* (see (5)) by using Lavenberg–Marquardt algorithm with Bayesian regularization described by Hagan et al. [24, 25].

(5) $$\begin{array}{c} E=\alpha \sum {\left|\;t_{i}-y_{i}\;\right|}^{2}+\beta E_{w}. \end{array}$$

Here, **y**_*i*_ is the output of the network at the *i*th data point, **t**_*i*_ is the target output of the *i*th data point and has the same structure as **y**_*i*_, *E*_*w*_ is the sum of squares of all the network weights and biases (i.e., **w**_*h*_, **b**_*h*_, **w**_*o*_, **b**_*o*_), and *α* and *β* are cost function parameters. The update laws of Bayesian optimization of regularization parameters *α* and *β* were described in [25]. Target output vector, **t**_*i*_, has three elements. The values of this vector represent the gait mode of walking. The meaning of the possible structures of the **t**_*i*_ vectors is illustrated in (6). 

*Three-Element Representation of Target Vector for Different Gait Modes.*

(6) tA=100⟹representsAscent  mode,tL=010⟹representsLevel  ground  mode,tD=001⟹representsDescent  mode,tX=000⟹representsUndetermined  mode.

#### 2.1.2. Collection of ANN Input

Detecting the input signals plays an important role for developing an algorithm for any task. In this study, our task was to recognize a change in gait mode. It has been observed that the sagittal plane rotation of the shank is different for level ground walking and stair walking [17]. From pilot data, we also found similar differences in foot segment rotation due to these different gait modes. Moreover, the vertical component of the velocity of the foot is also observed to be different for level ground, stair ascent, and descent modes [26]. For these reasons, we used the vertical velocity and segment angle of the foot shell of the PPAFO as input signals to our artificial neural network.

The vertical component of velocity and foot segment angle were calculated using an IMU sensor (XSens MTi-28A53G35; XSens Technologies; Enschede, The Netherlands; Figure 1). Before any calculation, the readings of the IMU were converted from IMU coordinates to earth coordinates for estimating the orientation of the IMU (see [16] for detailed procedure). For finding velocity, the obvious approach was to directly integrate the acceleration in the *z* direction. However, this approach became erroneous because of long term drifts. This long term drift can be avoided by recalibrating the velocity reading to zero at every zero-acceleration instance [16, 26]. Usually, the vertical component of the IMU acceleration reading should be *g* (*g* = 9.81 m/s^2^) when the foot segment is at rest during mid-stance phase. However, because of error in orientation of the signal and the input noise, we assumed that the zero-acceleration instance was achieved when equation (7) holds.

(7) $$\begin{array}{c} \left\|\;a_{z}-g\;\right\|<\varepsilon_{g}. \end{array}$$

**Figure 3:**
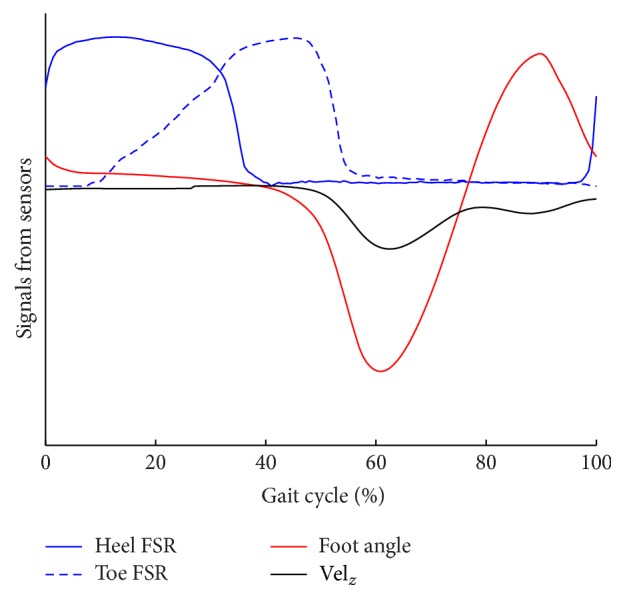
Different sensor signals during walking.

Here, *a*_*z*_ is the vertical component of acceleration in the world coordinate system collected from the IMU accelerometer and *ε*_*g*_ is the heuristically tuned threshold value found from training data so that the zero-acceleration instance will only occur during mid-stance during walking when the foot is in a stationary position. Due to noise during data collection, if this zero instance cannot be achieved via (7) for any step, the heel and toe force sensitive resistors (FSRs) can be used as a fail-safe to detect this event. That is, generally, during the zero-acceleration instance, both the heel and toe FSRs should be in “ON” states since they are in contact with the ground. For calculating the foot pitch angle (foot segment angle), data were collected in a quaternion based coordinate system. Using the equation found from the data sheet of the IMU, the foot segment angle was calculated. Thus the input signals to the ANN, vertical component of velocity and foot segment angle (Figure 3), were derived.

#### 2.1.3. Conditioning of ANN Output

**Figure 4:**
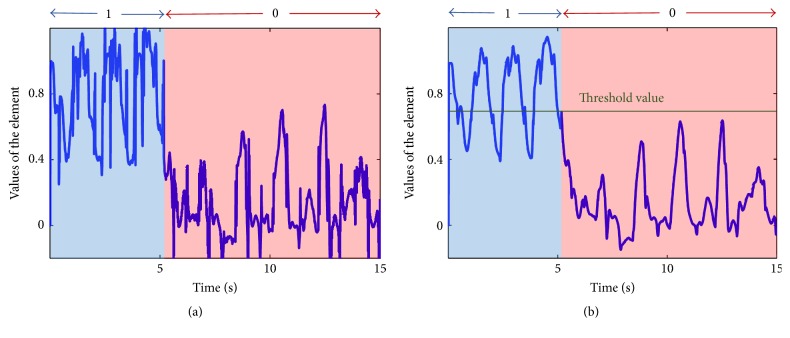
Example of the values of an element of output vectors **y** and $\bar{y}$. (a) Output of ANN before filtering (**y**); (b) output after filtering ($\bar{y}$).

(*1) Filtering the Output of the Network*. The output from the network **y**(*t*) was filtered to attenuate unwanted noise (Figure 4). **y** was passed through a first-order filter with time constant *ϵ* to obtain a new output variable $\bar{y}$. For this study, the time constant for the filter *ϵ* is chosen as 0.02 sec. The equation for the first-order filter was described in

(8) ϵy¯˙+y¯=y,y¯0=y0.

(*2) Conversion to Binary Values*. The filtered output $\bar{y}$ was used to detect gait modes. The values of each of the three elements of the output vector **y** are noninteger; thus, the elements of the filtered output $\bar{y}$ are also noninteger values (Figure 4). Each element was converted to either “0” or “1” (integer binary value) after establishing a threshold value. From the training data, the thresholding value for each element was calculated by minimizing the risk using a loss matrix as described below [27]. Although a simple step function where the 0-1 threshold was 0.5 could have been used, the statically chosen linear activation function based on the filter improved the accuracy of the algorithm.

Let us assume that there were two classes (*w*_1_ and *w*_2_) for each element of the filtered output vector. *w*_1_ represents the class when the value of the element should be 0 and *w*_2_ represents the class when the value of the element should be 1. Assume that *z* is the noninteger value of the element. *λ*_*ij*_ is a penalty term which is known as the loss that depends on the wrong decision. The loss matrix* L* [27] is defined by the following equation:

(9) $$\begin{array}{c} L=\left[\begin{array}{cc} \lambda_{11} & \lambda_{12}\\ \lambda_{21} & \lambda_{22} \end{array}\right]. \end{array}$$

Here *λ*_*ij*_ means the value of risk if an element of class *i* is classified as class *j*. As *λ*_11_ and *λ*_22_ represent the risk of classifying class 1 and class 2 correctly, respectively, these two values should be 0. We assumed that the element misclassifying class-1 has higher risk compared to misclassifying class-2 to be on the conservative side for detecting a gait mode. For this reason, we chose the value of risk of misclassifying class-1 (*λ*_12_) to be twice as that of misclassification of class-2 (*λ*_21_) to account for this conservative side. That means we chose

(10) $$\begin{array}{c} \lambda_{12}=2\lambda_{21}. \end{array}$$

So, we chose our loss matrix as follows:

(11) $$\begin{array}{c} L=\left[\begin{array}{cc} 0 & 1.0\\ 0.5 & 0 \end{array}\right]. \end{array}$$

We assumed that these two classes with the single element value *z* have Gaussian probability density functions.

(12) pz ∣ w1=12σ1πe−z−μ12/2σ12,pz ∣ w2=12σ2πe−z−μ22/2σ22.

Here, (*μ*_1_, *σ*_1_) and (*μ*_2_, *σ*_2_) are means and standard deviations of probability density functions, *p*(*z*∣*w*_1_) and *p*(*z*∣*w*_2_), respectively. According to [27], the threshold value *z*_*o*_ in this study can be found by solving the following equation:

(13) $$\begin{array}{c} z_{o}\text{: }\lambda_{12}p\left(\;z\;|\;w_{1}\;\right)=\lambda_{21}p\left(\;z\;|\;w_{2}\;\right). \end{array}$$

For this study, (*μ*_1_, *σ*_1_) and (*μ*_2_, *σ*_2_) for each element of the output vector can be found from the training data. Using these threshold values, each of the three elements of the vector ($\bar{y}$) was converted to either “0” or “1” and, thus, $\bar{y}$ will be converted to $\hat{y}$, which is a binary version of $\bar{y}$.

#### 2.1.4. Classification of Different Modes

The proposed approach to detect the gait modes was implemented on the testing data by using the parameters determined from the training data. Assume that **x**′ was the input vector for any instance. Using the network parameters (**w**_*h*_, **b**_*h*_, **w**_*o*_, **b**_*o*_, determined during training), the output **y**′ was calculated by (1)–(4). Each of the elements of the output vector was first filtered (see (8)) and later converted to a binary value to determine the vector ${\hat{y}}^{\prime }$ (by using the three thresholding values found from the training data). Thus using the final output vector, ${\hat{y}}^{\prime }$, the current gait mode was determined using the values for each element as given in equation (6).

(14) $$\begin{array}{c} \begin{array}{c} Current\;\;gait\;\;mode⟹\left\{\begin{array}{cc} if\;\;{\hat{y}}^{\prime }=t^{A}, & Ascend\;\;mode,\\ if\;\;{\hat{y}}^{\prime }=t^{D}, & Descend\;\;mode,\\ if\;\;{\hat{y}}^{\prime }=t^{L}, & Level\;\;ground\;\;mode,\\ if\;\;{\hat{y}}^{\prime }=t^{X}, & Undetermined\;\;mode. \end{array}\right. \end{array} \end{array}$$

The proposed algorithm did not rely on any specific information regarding stair height or ramp grade, since it only depended on the vertical velocity and angle of the foot segment which reflected an individual's walking behavior. For this reason, this algorithm should recognize the gait mode regardless of stair height or ramp grade.

### 2.2. Experimental Data Collection

Data collected in the previous study [16] were used to evaluate the proposed ANN based algorithm. In that study, five healthy male subjects (average age: 23.4 y, average weight: 82.0 kg, and height 178.6 cm) participated and gave informed consent. The study was approved by University of Illinois Institutional Review Board. The subjects wore the PPAFO on the right leg.

The detailed hardware description of the PPAFO can be found in [16, 28]. The PPAFO (Figure 1) had a pneumatic rotary actuator that could provide modest plantarflexor (~12 Nm) or dorsiflexor (~3 Nm) torque when powered using a small portable compressed carbon dioxide tank at 100 psig. In the current study, analyzed data were generated when the PPAFO was operated in passive mode with no ankle torque assistance. An embedded microcontroller (TMS320F28335, CPU: 150 MHz, Texas Instruments, Dallas, TX, USA) collected sensor signals from the IMU, two FSRs, and a rotary potentiometer for ankle angle. The potentiometer data were not used for gait mode recognition. The FSRs (#403, 2′′ square; Interlink Electronics Inc., Camarillo, CA, USA) were attached under the heel and ball of the foot between the foot shell and sole. The IMU was attached to the medial side of the foot shell. All signals were sampled at 200 Hz.

During a testing session, the subject walked with the PPAFO for two test scenarios and three trials per scenario. Two test scenarios were collected: walking on level ground and outdoor stairs and walking on level ground and an indoor ramp. The total height after traversing two steps was 28 cm (i.e., 14 cm per step rise). The ramp had a 6-degree grade. Specifically, for the stair scenario, subjects walked uninterrupted in the following order: (1) 3 to 4 steps on level ground, (2) 6 steps ascending stairs, (3) 3 to 4 steps on level ground, (4) turning back, (5) 3 to 4 steps on level ground, (6) 6 steps descending stairs, and (7) 3 to 4 steps on level ground. For the ramp scenario, the following order was used: (1) 3 to 4 steps on level ground, (2) 8 to 10 steps ascending the ramp, (3) turning back, (4) 8 to 10 steps in descending the ramp, and (5) 3 to 4 steps on level ground. For each scenario, the first trial was used to train the algorithm, and the other two trials were used to evaluate the performance of the proposed algorithm.

### 2.3. Data Analysis

**Figure 5:**
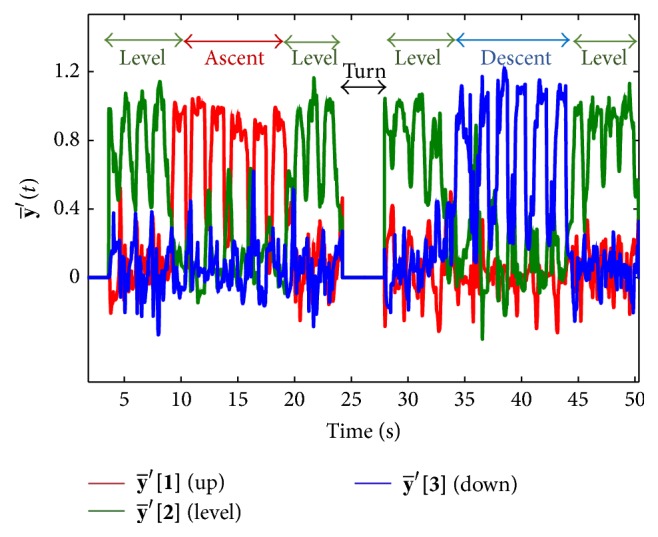
Output of the artificial neural network (after filtering) for different modes of walking.

Vertical velocity and foot segment angle data from the experimental trials were processed using the classification algorithm proposed above (in Section 2.1) and using the previous algorithm [16].  Figure 5 illustrates an example of filtered output of the artificial neural network. The red line represented the first element (${\overset{-}{y}}^{\prime }$ **[1]**) of the vector, ${\overset{-}{y}}^{\prime }$, which indicated ascent mode. Similarly green (indication of level mode) and blue (indication of descent mode) represented the second (${\overset{-}{y}}^{\prime }$ **[2]**) and third (${\overset{-}{y}}^{\prime }$ **[3]**) elements of the filtered output vector of the network, respectively (Figure 5). High values for an element signal were used to classify each step.

To understand how quickly each algorithm could detect a new gait mode, this time was computed from the start of the swing phase of the transition step and measured as a function of percentage of gait cycle (% GC) from the start of swing. One gait cycle was defined by consecutive heel strikes of the same limb and normalized into 0–100% GC.

Success rate was used to evaluate the performance of each algorithm for correctly identifying the gait mode of each step. Success rate was defined by the following [16]:

(15) Success  Rate=Number  of  Correctly  Recognized  StepsNumber  of  Total  Steps×100%.

The results of the newly proposed algorithm were compared with that of the algorithm described in [16].

## 3. Results

**Figure 6:**
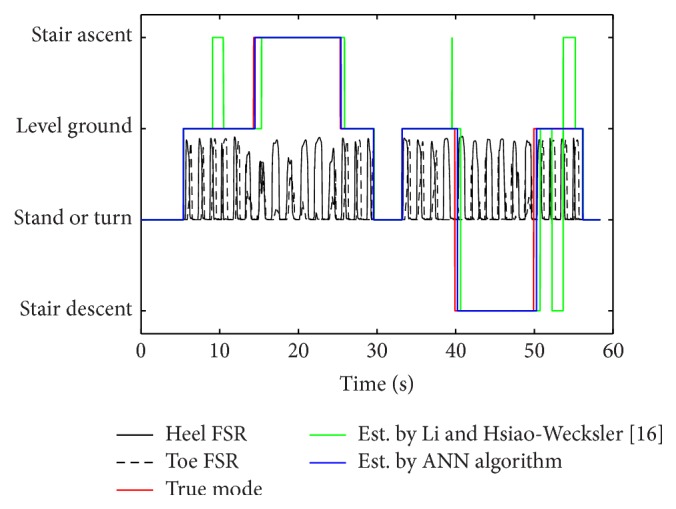
Comparison between true mode and mode estimated by original Li and Hsiao-Wecksler [16] and ANN algorithms.

After applying this approach, it was found that the proposed estimator can detect each mode more effectively compared to the previously developed algorithm [16] (Figure 6). The previous method had more incorrect step classifications. Additionally, the previous algorithm was not able to recognize the new mode until partway into the next step after the transition; thus resulting in a one-step delay. The proposed algorithm was able to recognize the new mode during the swing phase of the step during the transition. The proposed algorithm was able to detect a new gait mode within 28% GC, on average, after the start of the swing phase during the stair scenario (16% GC for ramp), whereas, with the previous algorithm, the new gait mode detected 77% GC after swing for the stair scenario (73% GC for ramp), which put this time into the stance phase of the next step.

Success rates for the proposed algorithm while walking during the stair scenario (Table 1) and ramp scenario (Table 2) were found to be larger compared to the results from the previous algorithm. Sub01 showed the best result for both algorithms (100%). However, success rates of other subjects had different values when compared between the two algorithms. Sub03 and Sub04 also had 100% success rate when the proposed algorithm was used. Overall, while subjects were walking during the stair scenario, success rates to detect level ground, ascent mode, and descent mode were 99.3%, 100.0%, and 98.3%, respectively. On the other hand, using the previous algorithm, these values were 93.4%, 98.3%, and 93.3%. Similar results were found when subjects walked during the ramp scenario.

## 4. Discussion

### 4.1. Experimental Observation

The primary purpose of this study was to develop an algorithm to automatically distinguish different gait modes (level ground, ascent and descent of stairs or ramps), as controller schemes for powered devices vary for different gait modes. When walking on level ground and ascending stairs or ramps, the ankle should be dorsiflexed and the toes held up to prevent tripping. When descending, the ankle should be plantarflexed and the toes point downward in preparation for contacting the lower surface. The proposed gait mode recognition algorithm, using an artificial neural network, successfully classified gait mode with high accuracy and without the previous one-step delay limitation. For both stair and ramp scenarios, the proposed algorithm demonstrated better performance compared to the algorithm developed by Li and Hsiao-Wecksler [16] (Tables 1 and 2).

For the stair scenario, the proposed algorithm worked very well in the ascent mode condition with an average success rate of 100%, while the previous vertical position tracking algorithm had an average success rate of 98.3% [16]. In level ground walking, the overall success rate was 99.3%, while previously it was 93.4%. The proposed algorithm had also shown better performance in descent mode in the stair scenario. Most of the time, except Sub02 for level ground mode and Sub05 for descent mode, the proposed algorithm's success rate was 100%. As a whole, when walking on stairs, the new algorithm performed better than the previous method based on success rate.

For the ramp scenario, the proposed algorithm had 98.9%, 97.8%, and 100% average success rate at level ground walking, ascent mode, and descent mode, respectively. All of these values are better than the corresponding values when the previous algorithm was used. For ramp descent mode, the proposed algorithm had the best result having 100% of success rate for all subjects. In the level ground mode, the proposed algorithm also had a 100% success rate, except for Sub01. Similarly, except for Sub03, the proposed algorithm had a 100% success rate. Overall, the new algorithm showed promising results while walking on the ramp.

The proposed algorithm used an artificial neural network where the inputs were a moving six-sample size window from the input signals: vertical velocity and foot segment angle. Since calculations were done continuously, this algorithm was faster at detecting a new gait mode, that is, soon after the new step was started. The average delay to detect a new gait mode was 28% GC and 16% GC after start of the swing phase for stair and ramp scenarios, respectively; whereas the previous algorithm was not able to detect a mode change until well into the next step. Additionally, because the signals from vertical velocity and angle of foot segment were used as input signals, the proposed algorithm should recognize the gait mode regardless of stair height or ramp grade. The previous algorithm depended on the height of the stair size or the grade of the ramp to make comparisons between threshold and real-time data, that is, the algorithm required new training to calculate thresholds for stairs with different heights and ramps with different grades.

### 4.2. Limitations and Future Recommendations

Though the proposed algorithm showed promising results, there are limitations of this study. One limitation of the proposed algorithm was that we used subject-specific training data. Ideally, a best approach will work for all wearers using a generic training data set. Making training data from multiple subjects or using input from multiple IMUs (e.g., one IMU on foot and another IMU on shank) might help to overcome this limitation. Furthermore, we used the success rate as the measurement of the performance of the algorithms. As mentioned in [16], it is not clear what should be the acceptable value of success rate [9, 13, 17, 29, 30].

There are several aspects of this study which open prospects for new studies. The current algorithm was developed and applied on previously collected data. Implementation of the algorithm to detect the gait mode and also control for ankle actuation should be addressed in the future. It was hypothesized that the proposed algorithm should not depend on stair height and the grade of the slope. A new study should check the claim. Training and testing were done separately for the two scenarios (stairs and ramps). A future recommendation would be to create a test protocol that mixed both scenarios during both testing and training to show greater flexibility to identify multiple environments. Overall, the current study demonstrated that an artificial neural network can be used to detect gait modes with higher accuracy and opened new opportunities for exploring the area of recognizing gait modes.

## 5. Conclusion

Portability of powered orthotic or prosthetic devices opened new challenges to detect gait modes (level ground, ascent and descent during walking on stairs or ramps). The actuation of these kinds of powered assistive devices should be changed accordingly based on the gait modes. Manually switching for a new gait mode is the most common approach. In this study, a novel algorithm based on an artificial neural network was proposed which continuously analyzed the input signals for automatically detecting the gait mode using an inertial measurement unit. This algorithm recognized new gait modes faster and with higher accuracy than a previous method used with the PPAFO.

## Figures and Tables

**Table 1:** Success rate during stair scenario.

	ANN algorithm Success rate (%)			Algorithm by Li and Hsiao-Wecksler [16] Success rate (%)		
	Level mode	Ascent mode	Descent mode	Level mode	Ascent mode	Descent mode
Sub01	100.0	100.0	100.0	100.0	100.0	100.0
Sub02	96.9	100.0	100.0	81.3	100.0	91.7
Sub03	100.0	100.0	100.0	100.0	100.0	91.7
Sub04	100.0	100.0	100.0	93.3	91.7	91.7
Sub05	100.0	100.0	91.7	93.3	100.0	91.7

Overall	99.3	100.0	98.3	93.4	98.3	93.3

**Table 2:** Success rate during ramp scenario.

	ANN algorithm Success rate (%)			Algorithm by Li and Hsiao-Wecksler [16] Success rate (%)		
	Level mode	Ascent mode	Descent mode	Level mode	Ascent mode	Descent mode
Sub01	95.0	100.0	100.0	90.0	100.0	94.7
Sub02	100.0	100.0	100.0	94.4	94.1	93.8
Sub03	100.0	90.0	100.0	94.4	90.0	95.0
Sub04	100.0	100.0	100.0	93.3	100.0	94.1
Sub05	100.0	100.0	100.0	87.5	100.0	93.8

Overall	98.9	97.8	100.0	92.0	96.7	94.3
